# Functional septorhinoplasty alters brain structure and function: Neuroanatomical correlates of olfactory dysfunction

**DOI:** 10.3389/falgy.2023.1079945

**Published:** 2023-04-06

**Authors:** Katherine L. Whitcroft, Laura Mancini, Tarek Yousry, Thomas Hummel, Peter J. Andrews

**Affiliations:** ^1^ UCL Ear Institute, University College London, London, United Kingdom; ^2^ Centre for the Study of the Senses, Institute of Philosophy, School of Advanced Study, University of London, London, United Kingdom; ^3^ Department of Rhinology and Facial Plastics, Royal National Throat Nose and Ear Hospital, London, United Kingdom; ^4^ Department of Otorhinolaryngology, TU Dresden, Dresden, Germany; ^5^ Lysholm Department of Neuroradiology, the National Hospital for Neurology & Neurosurgery, University College London Hospitals NHS Foundation Trust, London, United Kingdom; ^6^ Department of Brain Repair and Rehabilitation, UCL Queen Square Institute of Neurology, London, United Kingdom

**Keywords:** olfaction, olfactory dysfunction, grey matter volume, cortical thickness, functional MRI, plasticity, treatment, septorhinoplasty

## Abstract

**Introduction:**

We previously demonstrated functionally significant structural plasticity within the central olfactory networks, in association with improved olfaction after surgical treatment of chronic rhinosinusitis (CRS). In order to confirm and expand on these findings, the primary aim of this study was to determine whether these same regions undergo functionally significant structural plasticity following functional septorhinoplasty (fSRP), in patients with non-CRS olfactory dysfunction (OD) of mixed cause. fSRP has previously been shown to improve olfactory function, and the secondary aim of this study was to provide initial insights into the mechanism by which fSRP affects olfaction.

**Methods:**

We performed a pilot prospective, multimodal neuroimaging study in 20 participants undergoing fSRP, including patients with non-CRS OD of mixed cause, as well as normosmic surgical controls. Participants underwent psychophysical olfactory testing, assessment of nasal airway, structural and functional neuroimaging. This was performed pre- and postoperatively in patients, and preoperatively in controls.

**Results:**

There was a statistically and clinically significant improvement in mean psychophysical olfactory scores after surgery. This was associated with structural and functional plasticity within areas of the central olfactory network (anterior cingulate, orbitofrontal cortex, insula, temporal pole). Improved psychophysical scores were significantly correlated with change in bilateral measures of nasal airflow, not measures of airflow symmetry, suggesting that improved overall airflow was more important than correction of septal deviation.

**Conclusion:**

This work highlights the importance of these neuroanatomical regions as potential structural correlates of olfactory function and dysfunction. Our results also provide initial insight into the mechanistic effects of fSRP on olfaction. Further work could investigate the utility of these regions as personalised biomarkers of OD, as well as the role of fSRP in treating OD.

## 1. Introduction

Neuroanatomical correlates of olfactory function and dysfunction have been previously suggested through cross-sectional studies comparing patients with healthy controls (1–5). Longitudinal studies are, however, superior in attributing causality (6). In a previous multimodal prospective neuroimaging study, we demonstrated functionally relevant structural plasticity within the anterior cingulate cortex (ACC), insula, orbitofrontal cortex (OFC) and temporal poles (TP), in association with improved olfaction, after functional endoscopic sinus surgery (FESS) for chronic rhinosinusitis (CRS) (7). To both confirm and expand on these findings, and in particular to determine whether the changes demonstrated in these structures are aetiology and treatment specific, the primary aim of the present study was to characterise structural and functional plasticity of these regions in response to functional septorhinoplasty (fSRP), in patients with non-CRS olfactory dysfunction (OD) of mixed cause.

fSRP has previously been shown to improve olfaction, though the relevant evidence base is limited by methodological inconsistencies (8). Given the paucity of currently available treatment options for OD, our secondary aim was to gather pilot data investigating the potential mechanism by which fSRP improves olfaction, using subjective, psychophysical, and for the first time, more objective structural and functional neuroimaging measures.

We therefore performed a pilot prospective, multimodal neuroimaging study [voxel-based morphometry (VBM), analysis of cortical thickness and olfactory functional MRI] in patients with non-CRS OD of mixed cause undergoing fSRP, compared with a normosmic preoperative surgical control group. We hypothesised that improved olfactory function will be accompanied by increased BOLD signal and structural change within the ACC, insula, OFC and TP. Mechanistic insight into how fSRP affects olfaction could provide proof of concept for further work investigating its clinical utility as a potential treatment for OD. Moreover, where our results replicate those demonstrated in our CRS cohort, this will help to confirm these regions as neuroanatomical correlates of general, rather than disease specific OD.

## 2. Materials and methods

We performed a prospective cohort study in patients (18–70 years) with OD undergoing fSRP to improve nasal airflow. Patients were only eligible for the study if they both had established OD *and* required fSRP, limiting the available (pre-pandemic) patient population. Therefore, a pragmatic study sample was used and patients with OD (defined as TDI score of <30.75—see below) of mixed aetiology were included. Patients with OD due to head injury or suspected/confirmed neurodegenerative disease were excluded, due to potential baseline structural brain alterations, as were those with CRS [diagnosed by the senior author based on the contemporaneously available EPOS 2012 guidelines (9): clinical history, examination findings including endoscopy, and imaging]. Patients with allergic rhinitis were only included where their OD persisted despite full medical management according to the EPOS 2012 guidelines (9), and where it was possible to exclude CRS based on careful examination of clinical, endoscopic and imaging findings. We additionally excluded patients who were not available for follow-up testing, or those who had contra-indications to MRI scanning. As handedness does not appear to affect passive olfactory processing (10), patients who were otherwise clinically eligible were not excluded based on handedness alone. Normosmic control participants were taken from the same population of patients awaiting fSRP (“surgical controls”), in an effort to ensure the groups were otherwise comparable and reduce the effect of confounding factors. Controls were age/sex matched, with other exclusion criteria as per patients. All subjects were asked to refrain from smoking, eating or drinking (except water) for 1 h prior to their assessment session.

**Figure 1:**
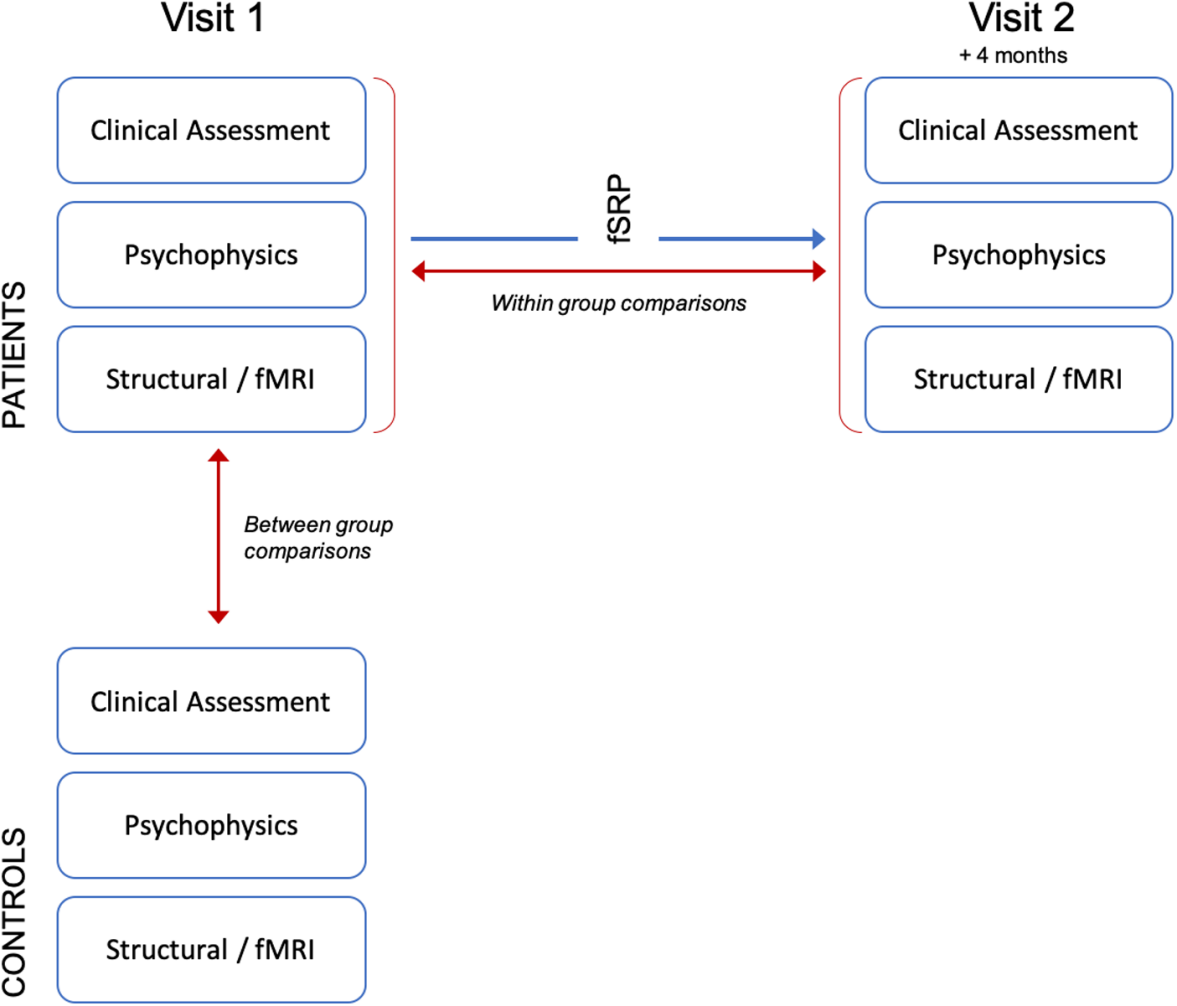
Experimental paradigm.

All participants underwent clinical assessment, psychophysical testing and neuroimaging. In patients, this was performed at baseline (visit 1), and again at 4 months post-operatively (visit 2, see Figure 1). Controls were assessed at baseline only. Clinical assessment included thorough medical history taking (including duration of OD) and completion of patient reported outcome measures “SNOT23” (11), “NOSE” score (12) and VAS (13). Clinical examination included three-pass rigid nasendoscopy and bilateral peak nasal inspiratory flow rate (PNIF). In the patient cohort unilateral PNIF measurements were collected at visit 1 and 2 in order to determine change in symmetry of nasal airflow after surgery. These were used to calculate two scores, with the aim of directly measuring the functional significance of septal deviation [where “*R*” and “*L*” = unilateral PNIF flow rate for right and left side. PNIF values of <30l/min assigned 0]: (1) The absolute difference in airflow between right and left nostrils in L/min (“*AD*”); (2) Airflow symmetry (“*AS*”)—where 0 denotes equal airflow between right and left sides, and values closer to 0 indicate greater symmetry.

AD=|R−L|

$$AS\;=\left|\frac{R}{R+L}\;-0.5\right|$$

Psychophysical olfactory testing was performed using the Sniffin’ Sticks tool [a measure of composite (TDI) and individual odour threshold (T), discrimination (D) and identification (I)—for detailed description of testing procedure, see (14)]. To reduce participant burden in our clinical cohort, and to assess olfactory function in a way that is functionally relevant to patients, we elected to perform olfactory testing/fMRI birhinally. Normosmia was attributed where TDI was ≥30.75, hyposmia where TDI is >16, but <30.75, and functional anosmia ≤16 (15). The minimal clinically important difference (MCID) for T, D and I are ≥2.5 points, ≥3 points and ≥3 points respectively, and ≥5.5 points for composite TDI (16).

MRI scans were also used to calculate Lund-Mackay (LM) scores for both patient and control groups [where mean “normal” score in patients without CRS = 4.3 (17)].

**Figure 2:**
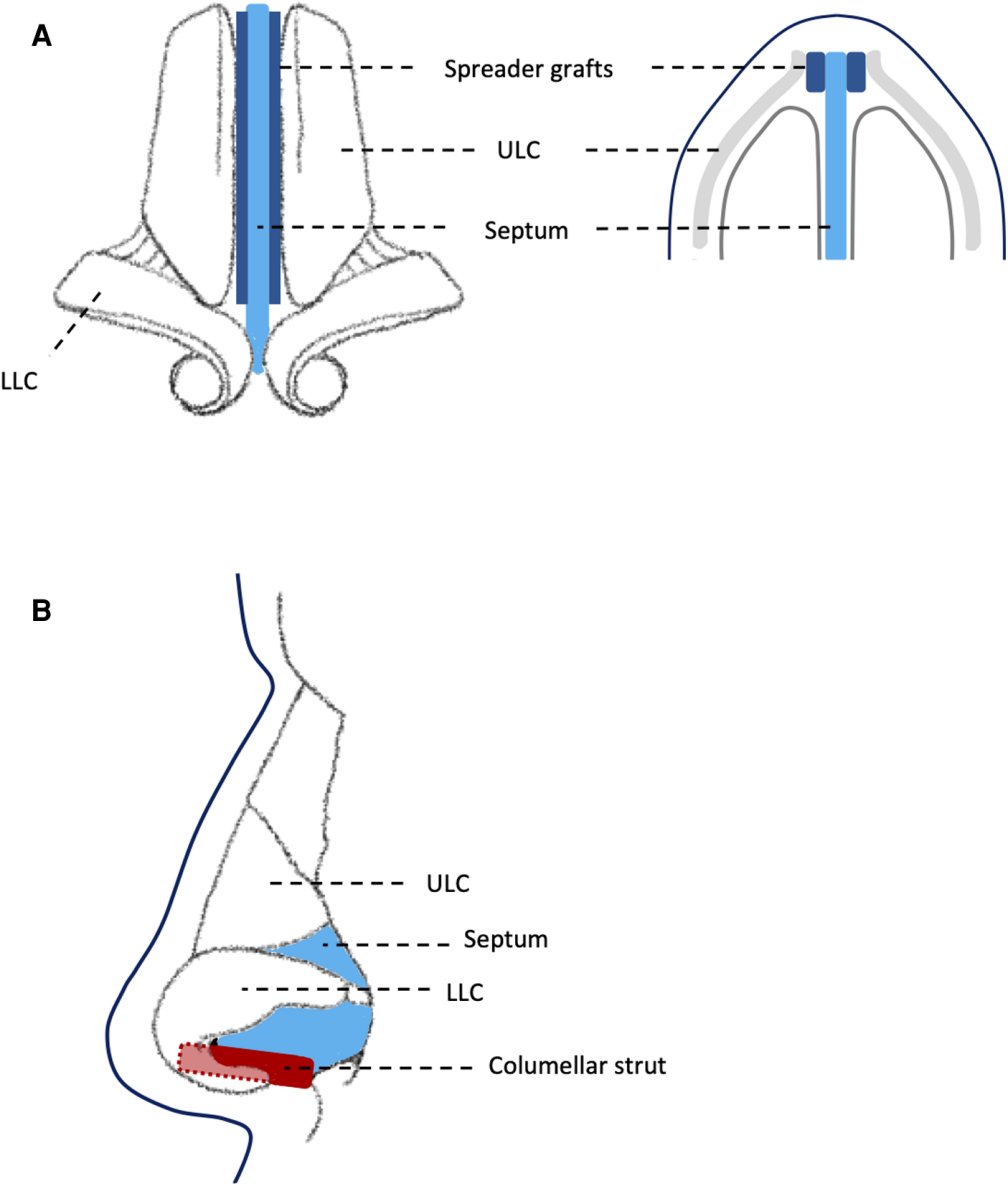
Diagram showing: (**A**) standardised placement of spreader grafts between septum and upper lateral cartilage; (**B**) standardised placement of columellar strut between medial crura of lower lateral cartilage. ULC, upper lateral cartilage; LLC, lower lateral cartilage.

All patients underwent fSRP using a standardised external approach, aiming to maximise symmetrical, bilateral nasal airflow. This involved three main stages: (1) septoplasty with nasal bone realignment, increasing airway symmetry; (2) internal nasal valve augmentation using spreader grafts (autologous cartilage), increasing width of nasal airway; (3) external valve augmentation using columellar strut (autologous cartilage), increasing height of nasal airway. Figure 2 illustrates standardised placement of spreader grafts and columellar struts.

### 2.1. Functional MRI paradigm

All participants underwent olfactory functional MRI, in addition to structural imaging. Two odorants were used for functional imaging (one per functional run): banana (neat, aroma, Dale Air, Rochdale, UK) and cis-3-hexenol (neat, single molecule with smell of cut grass, Firmenich, Middlesex, UK). These were shown in pilot work to be iso-intense. During each run, a single odorant was presented birhinally in a block design. During “on” blocks, odours were delivered in 1-second pulses, embedded in 1l/min clean humidified air, with a 2-second interstimulus interval. During “off” blocks, clean humidified air only was delivered. Odorants were delivered to participants *via* Teflon® nasal cannulae (4 mm internal diameter) and through use of a computer controlled olfactometer (18). Due to low flow rates (which do not produce perceptible thermo-mechanical trigeminal activation), warming was not required.

**Figure 3:**
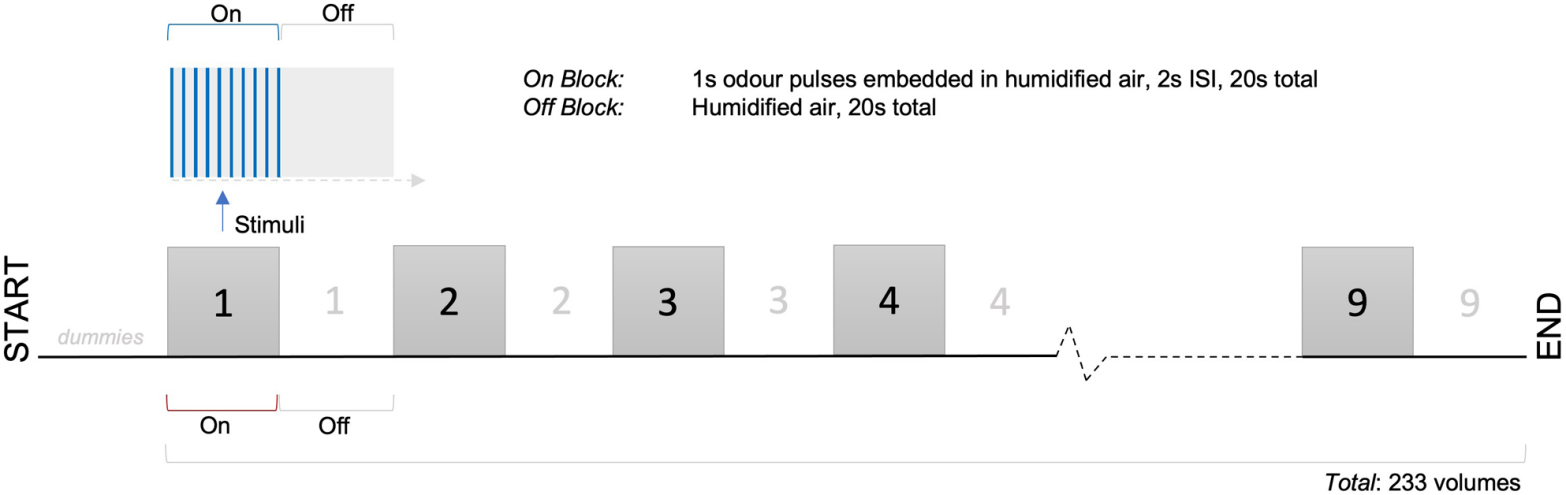
fMRI experimental paradigm.

On and off blocks were of duration 20s. There were 9 on and 9 off blocks, with 233 volumes in total. Each participant underwent two functional runs per scanning session, with order of first odour pseudorandomised and counter-balanced across participants. At the end of each functional run, participants were asked to rate odour intensity (0–10, 10 = strongest) and hedonic valence (−5 to +5, +5 = most pleasant). See Figure 3 for schematic diagram of experimental paradigm.

### 2.2. Imaging acquisition

Whole brain MRI was performed using a 3-T scanner (MAGNETOM Prisma, Siemens, Erlangen, Germany) with 64-channel head coil. Sagittal T1-weighted images were acquired using a 3-dimensional magnetization-prepared rapid acquisition gradient echo (MPRAGE) sequence. The following parameters were used: repetition time (TR), 2,000 ms; echo time (TE), 1.96 ms; inversion time (TI), 880 ms; field of view (FOV), 282 mm × 282 mm; matrix size, 256 × 256; one slab, 208 slices per slab; voxel size, 1.1 mm × 1.1 mm × 1.1 mm; and flip angle, 8°. Functional data were collected using a 2D GE-EPI sequence, TR 1,550 ms, TE 26 ms, FOV 200 mm, FA 75°, voxel size 2.5 mm × 2.5 mm × 2.5 mm (in total, 50 slices).

### 2.3. Imaging analysis: voxel based morphometry

Voxel based morphometry was performed using the CAT12 toolbox (available from http://dbm.neuro.uni-jena.de/vbm/) implemented in SPM12 (Wellcome Centre of Imaging Neuroscience, UCL, London, United Kingdom) and MATLAB (The MathWorks, Natick, MA, United States). All T1 images were visually inspected and reoriented as required according to SPM priors, and checked for obvious artefact. Images were then segmented into grey matter (GM), white matter (WM) and cerebrospinal fluid (CSF). For longitudinal patient images this was done using the longitudinal segmentation tool. This process involves an initial intra-subject inverse-consistent spatial realignment with bias correction between the pre-operative and post-operative images. In addition to segmentation of images from each time point, a mean image across time points is produced. Estimated spatial normalisation parameters were then calculated for the segmented mean image, using Diffeomorphic Anatomical Registration Through Exponentiated Lie Algebra (DARTEL) (19). The resultant DARTEL deformations are then applied to the segmented images at each time point, prior to their modulation. Images were then smoothed using a Gaussian kernel (FWHM, 8 mm). For comparisons between patients and controls, pre-op patient and control GM segmentations were also spatially normalised using DARTEL, modulated and smoothed (FWHM, 8 mm). Automated data quality checks were performed as per the CAT12 toolbox. Significant voxels are reported in relation to the Montreal Neurological Institute (MNI) coordinate space.

Differences in GM volume between patients and controls was compared using a two sample *T* test, controlling for total intracranial volume [“TIV”, summated GM, WM and CSF volume (19)], age and sex. A within group comparison to determine GM volume change after surgery in patients was also performed, using a flexible factorial model at the second level, with the between subject factor = subject (1 level: patients) and the within subject factor = time (2 levels: first scan, second scan), controlling for TIV. *T* tests for significant increase and decrease in GM volume between visits were performed. An absolute threshold masking value of 0.1 was applied to avoid possible edge effects between different tissue types (1, 3, 20).

In order to further investigate potential associations between change in psychophysical score and change in GM volume, beta weights were extracted from clusters of significant GM volume change demonstrated during the above within group analysis. As psychophysical scores were not used to identify these clusters, circular analysis was avoided. Extracted beta weight values were used to test for significant correlation between change in GM volume (ΔGM volume = second scan—first scan) and change in psychophysical score (ΔT/I/TI = post-op score—pre-op score). Results were thresholded using a *P* value that was Bonferroni corrected for multiple comparisons.

As we were particularly interested in plastic change within the areas identified in our previous work—the ACC, insula, OFC and TP—we performed a region of interest (ROI) analysis, in addition to whole brain analysis. The *a priori* ROIs were constructed within the WFU_PickAtlas software (available from: http://fmri.wfubmc.edu/software/pickatlas), based on the Automated Anatomical Labeling (AAL) atlas (21). The OFC ROI was constructed as per Kahnt et al. and therefore included the bilateral AAL regions of: superior, middle, inferior and medial orbital gyri as well as the rectal gyri (22). All whole brain analyses were corrected for multiple comparisons at the family wise error level (*P *< 0.05_FWE_). For the *a priori* ROI analysis, small volume corrections were implemented through the “ROI” function in WFU_PickAtlas and results were further corrected for multiple comparisons at the FWE level (*P *< 0.05_FWE_), or at an uncorrected threshold of *P *< 0.001_uncorr_. For exploratory purposes, we additionally used a more lenient Bonferroni corrected *P* value: *P *= 0.05/[number of ROI × 2_Right + Left_] = 0.05/8 = 0.00625. In order to avoid issues surrounding non-stationarity in voxel based volumetric analysis (23) we report only voxel based results.

### 2.4. Imaging analysis: cortical thickness

Cortical thickness was analysed using CAT12. Patient and control T1 weighted images were initially segmented using the surface and thickness estimation writing options. This uses a projection-based thickness approach to determine CT by estimating WM distance, and then projecting the local maxima onto other GM voxels. The latter is done using a neighbour relationship that is defined by the WM distance. The local maxima are therefore equal to the cortical thickness. During longitudinal segmentation, as in the VBM pipeline, in addition to segmentation of images from each time point, a mean image across time points was produced. Estimated spatial normalisation parameters were calculated for the segmented mean image and applied to the first and second images. Resultant surface data from both the right and left hemispheres were then smoothed using a 15 mm FWHM kernel.

Change in CT was compared between groups (patient vs. control) and within groups (patient preoperative vs. postoperative), as for VBM analysis. All CT analyses were performed at the whole brain level, with results thresholded at *P *< 0.05_FWE_.

### 2.5. Imaging analysis: functional MRI

Functional data was again analysed using SPM12. Anatomical T1-weighted images were inspected and reoriented according to SPM priors during VBM analysis. Functional images were additionally visually inspected for correct orientation according to SPM priors. Pre-processing involved initial realignment and unwarping of functional images followed by segmentation of T1-weighted images according to SPM tissue probability maps. Co-registration of functional and anatomical images was then performed, as well as normalisation to MNI space. Finally, data were smoothed using an 8 mm FWHM kernel. We then performed a first level analysis in which the condition “odour > baseline” was modelled for each subject, using the canonical haemodynamic response function. Resultant contrast images were then subjected to a second level random-effects analysis. Second level between and within group analyses were performed as for structural analyses. We additionally performed a second level regression analysis in order to test for positive correlations between psychophysical test score and BOLD signal, across all scans, correcting for age, sex and group (patient_visit 1, patient_visit 2, control). Whole brain analyses were corrected for multiple comparisons at *P *< 0.05_FWE_. *A priori* ROI analysis (with small volume correction) was conducted as per structural work, with results thresholded at *P *< 0.05_FWE_, *P *< 0.001_uncorr_ or the exploratory *P *< 0.00625. As non-stationarity is not an issue in functional analysis, we additionally used cluster-based inference for the latter two lenient thresholds, and only report clusters of ≥10 voxels.

Images for inclusion in the manuscript were prepared using the Xjview toolbox for SPM (available from: http://www.alivelearn.net/xjview/) and Microsoft PowerPoint.

### 2.6. Statistical analysis of non-neuroimaging data

Data for descriptive statistics were analysed using GraphPad Prism (version 6, GraphPad Software, LaJolla, United States). Unless specified otherwise, statistical significance was attributed where *P *< 0.05 and data are given as mean (SD) for parametric data or median for non-parametric.

### 2.7. Compliance with ethical standards

This study received NHS ethical approval (REC ref 14/SC/1180) and was conducted in accordance with the Declaration of Helsinki. All participants provided full informed written consent prior to participation.

## 3. Results

### 3.1. Demographics, behavioural and clinical scores

Twenty participants were initially recruited: ten patients (three PIOD, seven idiopathic OD) and ten controls. One patient was lost to follow up (PIOD). Clinical information regarding septal deformity and surgical procedure is provided in Table 1. T1-weighted images were available from all participants. Functional images from one patient (preoperative visit) and one control were excluded from analysis due to breath holding/movement artefact. Patient demographics, behavioural and clinical scores are shown in Table 2. There were no reported surgical complications or known requirements for revision within the patient group.

**Table 1:** Clinical deformity and surgical procedure.

Patient	OD aetiology	Deformity (Cottle's classification)	Procedure
1	IOD	DNS L, III	Septoplasty, NB realignment, spreader grafts, columellar strut
2	IOD	DNS L, II/III	Septoplasty, NB realignment, spreader grafts, columellar strut
3	IOD	DNS L, I/II DNS R, III	Septoplasty, NB realignment, spreader grafts, columellar strut
4	PIOD	DNS L, I/II/III	Septoplasty, NB realignment, spreader grafts, columellar strut
5	PIOD	DNS L, III	Septoplasty, NB realignment, spreader grafts, columellar strut
6	IOD	DNS L, I/II DNS R, III/IV	Septoplasty, NB realignment, spreader grafts, columellar strut
7	IOD	DNS L, III/IV	Septoplasty, NB realignment, spreader grafts
8	IOD	DNS L, I/II DNS R, III	Septoplasty, NB realignment, spreader grafts, columellar strut
9	IOD	DNS R, II/III	Septoplasty, NB realignment, spreader grafts, columellar strut

DNS, deviated nasal septum; L, left; R, right; IOD, idiopathic olfactory dysfunction; PIOD, post-infectious olfactory dysfunction; NB, nasal bones.

**Table 2:** Group average demographic, nasal airflow symmetry, psychophysical and clinical scores in patients and controls, shown as mean (SD) or median.

	Patients vs. controls (visit 1)			Patients: pre- vs. postoperative (visit 1 vs. visit 2)		
	Patients *n* = 10	Controls *n* = 10	Patient vs. controls	Preoperative *n* = 9	Postoperative *n* = 9	Pre vs. postoperative
Demographics						
Age, years	35.8 (12.3)	38.1 (13.0)	Fisher's Exact *P *> 0.99	-	-	-
Sex (M:F)	8:2	8:2	Fisher's Exact *P *> 0.99	-	-	-
Duration OD, yrs	6 (3)	-	-	-	-	-
Allergic Rhinitis (no of participants)	5	4	Fisher's Exact *P *> 0.99	-	-	-
Psychophysical olfactory scores						
T	3.3 (2.01)	8.5 (2.0)	*t*_18 _= 5.88, *P *< 0.0001*	3.3 (2.13)	5.08 (2.96)	*t*_8 _= 2.04, *P *= 0.076
D	7.30 (3.34)	12.4 (1.65)	*t*_18 _= 4.34, *P *= 0.0004*	7.22 (3.53)	9.44 (2.87)	*t*_8 _= 1.66, *P *= 0.14
I	7.0 (4.08)	13.0 (1.16)	*t*_18 _= 4.47, *P *= 0.0003*	7.0 (4.33)	9.44 (3.61)	*t*_8 _= 2.35, *P *= 0.047*
TDI	17.60 (8.20)	33.93 (1.83)	*t*_18 _= 6.14, *P *< 0.001*	17.47 (8.69)	23.97 (8.55)^c^	*t*_8 _= 2.55, *P *= 0.034*
Clinical scores						
SNOT-23	52.4 (22.74)	42.3 (25.02)	*t*_18 _= 0.85, *P *= 0.41	50.13 (25.2)	20.75 (17.50)	*t*_8 _= 2.99, *P *= 0.02*
SNOT-23: Olfaction	3.6 (1.27)	2.3 (1.83)	*t*_18 _= 1.85, *P *= 0.08	4^†^	2.65 (2.0)	*W *= −4.0, *P *= 0.50
VAS: Olfaction	8.45 (1.12)	4.13 (2.54)	*t*_18 _= 4.93, *P *= 0.0001*	8.3 (1.1)	5.25 (3.8)	*t*_8 _= 2.70, *P *= 0.031*
NOSE	59.5 (31.3)	68 (22.5)	*t*_18 _= 0.74, *P *= 0.47	58.1 (33.2)	31.3 (29.7)	*t*_8 _= 1.81, *P *= 0.11
LM	2^†^ [mean 2.7 (2.71)]	1^†^ [mean 2.9 (3.04)]	*U *= 45.5, *P *= 0.75	1^†^ [mean 3.22 (3.03)]	1^†^ [mean 2.89 (3.14)]	*W *= −2, *P *= 0.75
PNIF (Bilateral)	94.0 (44.4)	99.0 (46.77)	*t*_18 _= 0.25, *P *= 0.81	92.2 (46.7)	102.8 (34.8)	*t*_8 _= 0.60, *P *= 0.56
Nasal airflow symmetry scores						
AD	-	-	-	45.6 (40.3)	18.9 (15.4)^a^	*t*_8 _= 1.87, *P *= 0.099
AS	-	-	-	0.17^†^ [mean 0.26 (0.23)]	0.06^†^ [mean 0.11 (0.15)]^b^	*W *= −22, *P *= 0.15

^a^ At individual level, improvement seen in 5 patients.

^b^ At individual level, improvement seen in 5 patients.

^c^ At individual level, clinically significant improvement seen in 5 patients.

*Denotes statistically significant results.

^†^ Median values.

At visit 1, there was a statistically and clinically significant difference in TDI score between patient and control groups, with the group mean falling within the hyposmic range for patients. After surgery, there was a statistically and clinically significant increase in group mean TDI in patients, with clinically significant improvements in TDI in five individuals (see Table 2).

**Figure 4:**
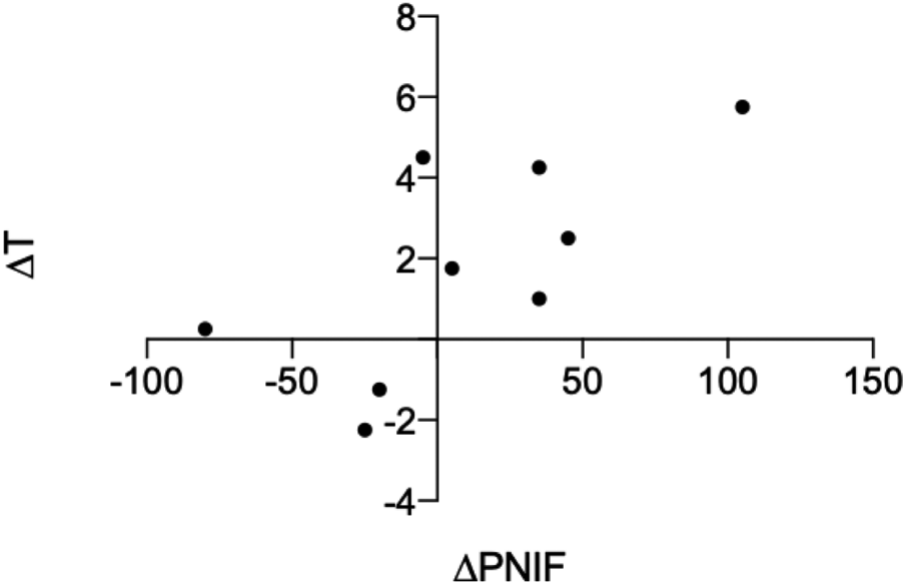
Significant positive correlation between change in PNIF and change in T score after surgery, *r* = 0.68, *P *= 0.04.

There were no statistically significant correlations between improved psychophysical test score (T/D/I/TDI) and LM score. There were no statistically significant correlations between change in *AD* or change in *AS* and change in psychophysical test score (T/D/I/TDI) after surgery. There was, however, a significant positive correlation between ΔT and ΔPNIF (bilateral) (*r* = 0.68, *P *= 0.04) (see Figure 4).

### 3.2. Voxel based morphometry

#### 3.2.1. Patients vs. controls

**Figure 5:**
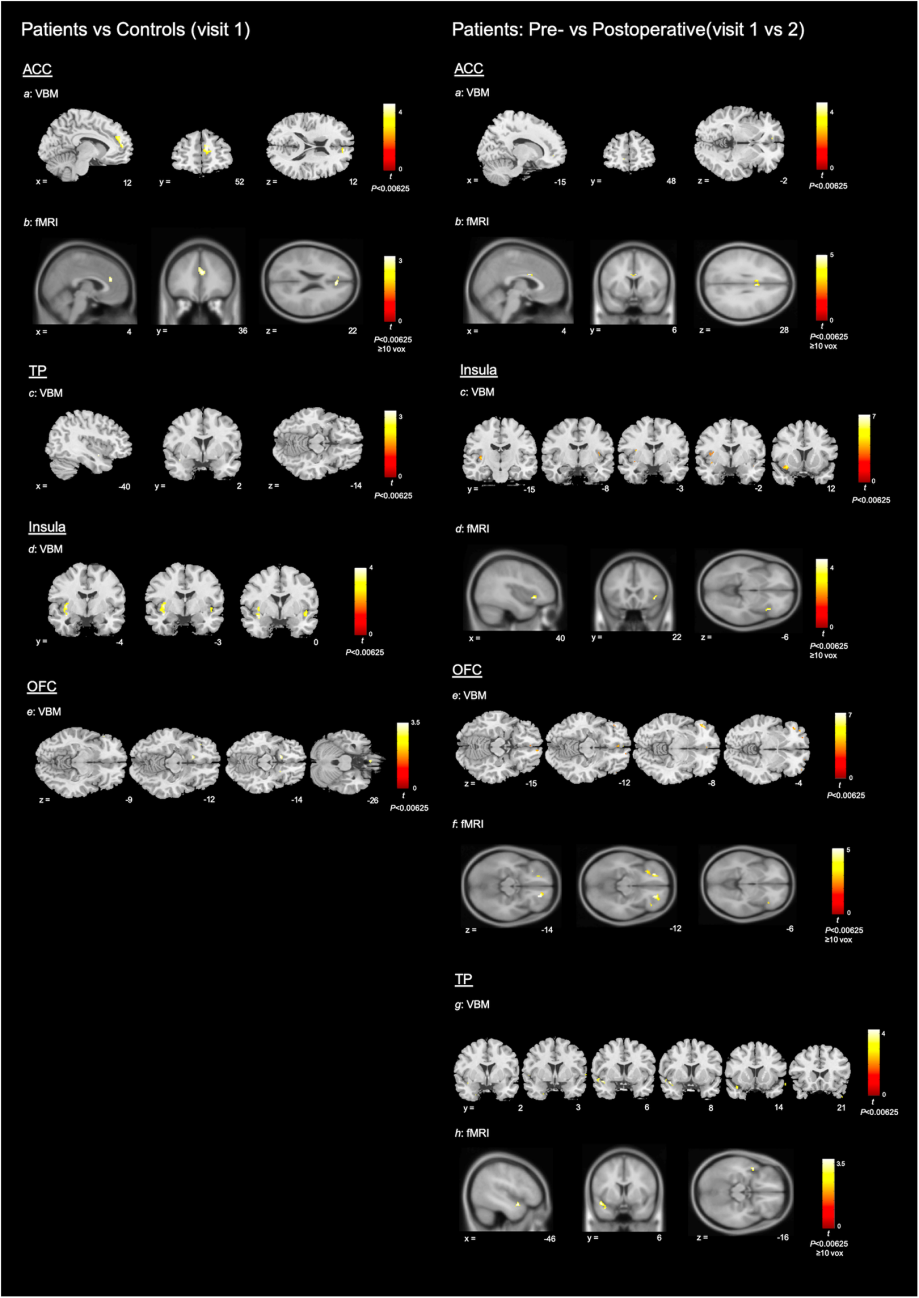
Neuroimaging results. To help differentiate between imaging modalities, VBM results are shown using the ch2bet stripped skull brain template and fMRI results are shown using the avg152T1 brain template. All coordinates are in MNI space. Colour bars show associated peak T score (please note that the maximum integer labelled may not reach top of colour bar range). **Left**: patients vs. controls (visit 1): Structural and functional MRI results for patients (visit 1) vs. controls. For ease of display—axial sections only shown for OFC results and coronal sections only shown for results within the insula. Subsections: (**a**) VBM results for increased GM volume within the ACC of patients compared with controls (*P *< 0.00625); (**b**) fMRI results for increased BOLD signal with the ACC of controls compared with patients (*P *< 0.00625, ≥10 voxels); (**c–e**) VBM results for increased GM volume in patients compared with controls, within the TP, insula and OFC (*P *< 0.00625). **Right**: Patients pre- vs. postoperative (visit 1 vs. 2): Structural and functional MRI results for decrease in GM volume and increase in BOLD signal after surgery, in the patient group. For ease of display—axial sections only shown for OFC results and coronal sections only shown for VBM results within the insula and TP. Subsections: (**a**) VBM results for decreased GM volume within the ACC of patients after surgery (*P *< 0.00625); (**b**) fMRI results for increased BOLD signal within the ACC of patients after surgery (*P *< 0.00625, ≥10 voxels); (**c–h**) VBM results for decreased GM volume and fMRI results for increased BOLD signal within the insula, OFC and TP of patients after surgery.

During *a priori* ROI analysis, we found small areas of decreased GM volume within the bilateral OFC, but more widespread areas of increased GM volume within each of the interrogated regions, in patients compared to controls (see Table 3A and Figure 5).

**Table 3A:** Voxels of significant GM volume difference between patients and controls (visit 1).

VBM results						
Patients vs. controls (visit 1)						
***ROI analysis***						
			MNI coordinates			
Threshold	ROI	Side	X	Y	Z	*T* Score
***Patient < control***						
*P *< 0.00625	OFC	L	−36	22	−20	2.99
		R	45	42	−21	2.87
***Patient > control***						
*P *< 0.001	ACC	R	12	52	12	4.61
	Insula	L	−38	−3	−14	4.01
		L	−38	−4	3	3.75
		L	−36	−3	0	3.74
*P *< 0.00625	Insula^†^	R	44	0	−8	3.35
	OFC	L	−14	14	−14	3.50
		L	−4	27	−26	3.08
		L	−54	27	−9	3.01
		R	20	12	−14	2.99
		L	−39	32	−12	2.89
	TP	L	−40	2	−14	3.33
***Whole brain analysis***						
No suprathreshold voxels						

Patients < controls indicates area of decreased GM volume in patients. Patients > controls indicates areas of increased GM volume in patients.

^†^ Results reported to demonstrate bilaterality at this lenient threshold—as left sided results significant at *P *< 0.001, only right sided results shown.

#### 3.2.2. Change in GM volume after surgery

Potentially in line with our preoperative between group findings, we demonstrated more widespread areas of decreased GM volume than increased GM volume after surgery, within the *a priori* ROIs (see Table 3B and Figure 5).

**Table 3B:** Voxels of significant change in GM volume after surgery, within patient group (visit 1 vs. 2).

VBM results						
Patients, pre- vs. postoperative (visit 1 vs. visit 2)						
***ROI analysis***						
			MNI coordinates			
Threshold	ROI	Side	X	Y	Z	*T* Score
***Increased GM volume***						
*P *< 0.00625	OFC	R	36	30	−20	3.58
***Decreased GM volume***						
*P *< 0.001	ACC	L	−15	48	−2	4.71
	Insula	L	−33	12	−12	7.27
		L	−38	−15	4	4.69
		L	−34	−3	14	4.65
	OFC	L	−45	39	−8	7.24
		L	−4	54	−12	5.20
		L	−39	51	−4	4.99
		R	6	57	−15	4.70
*P *< 0.00625	Insula^†^	R	39	−8	8	4.04
		R	42	−2	−14	3.44
	TP	L	−44	8	−14	4.30
		L	−54	6	−8	3.61
		L	−24	2	−36	3.58
		R	60	14	−15	3.52
		L	−54	3	0	3.50
		L	−40	2	−14	3.36
		R	44	21	−38	3.31
		R	62	3	2	3.31
***Whole brain analysis***						
No suprathreshold voxels						

Results are only shown at more lenient thresholds where none survive at *P *< 0.05_FWE_ or *P *< 0.001 as applicable.

^†^ Results reported to demonstrate bilaterality at this lenient threshold—as left sided results significant at *P *< 0.001, only right sided results shown.

#### 3.2.3. Correlation between change in psychophysical score and change in GM volume

There were no correlations between ΔGM volume (from 19 clusters of significant GM change as outlined in Table 3) and Δpsychophysical score that were statistically significant at the specified results threshold of *P *< 0.0026 [Bonferroni corrected *P *< 0.05/19].

### 3.3. Cortical thickness

No results survived thresholding during between (preoperative patient vs. control) or within (patient group: before vs. after surgery) group analyses at the whole brain level (*P *< 0.05_FWE_).

### 3.4. Functional MRI

There was no statistically significant difference in perceived intensity or hedonic valence for the two odour stimuli within the patient group, at visit 1 or 2 (see Table 4). Similarly, there was no significant difference in perceived intensity or hedonic valence for the two odours within the control group. Further analysis of the conditions “banana” and “grass” were therefore pooled.

**Table 4:** Intensity and hedonic ratings (valence) of fMRI odours, shown as mean (SD) or median values.

	Patients								Controls		
	Visit 1			Visit 2			Visit 1 vs. visit 2		Visit 1		
	Banana	Grass	Banana vs. grass	Banana	Grass	Banana vs. grass	Banana	Grass	Banana	Grass	Banana vs. grass
Intensity	3.44 (3.17)	2.94 (2.78)	*t*_8 _= 0.55, *P *= 0.60	4.44 (3.47)	4.94 (3.21)	*t*_8 _= 1.029, *P *= 0.33	*t*_8 _= 1.50, *P *= 0.17	*t*_8 _= 2.77, *P *= 0.024*	6.5 (0.87)	6.39 (2.15)	*t*_8 _= 0.22, *P *= 0.83
Valence	1.06 (1.84)	0.0^†^	*W *= −11, *P *= 0.28	1.50 (2.21)	0.11 (2.32)	*t*_8 _= 1.17, *P *= 0.27	*t*_8 _= 0.50, *P *= 0.63	*t*_8 _= 0.45, *P *= 0.67	3.11 (2.32)	0.67 (2.73)	*t*_8 _= 1.80., *P *= 0.11

^†^ Median values.

*Indicates statistically significant result.

#### 3.4.1. Patients vs. controls

Increased functional activity was demonstrated within the ACC of controls, compared with patients, during *a priori* ROI analysis (cluster peak originating to right but extending across midline) (see Table 5A and Figure 5).

**Table 5A:** Cluster of increased BOLD signal in controls, compared with patients (visit 1).

fMRI results							
Patients vs. controls (visit 1)							
***ROI analysis***							
			MNI coordinates				
Threshold	ROI	Side	X	Y	Z	*T* Score	*k*
*P *< 0.00625, 10 voxels	ACC	R^†^	4	36	22	3.22	78
***Whole brain analysis***							
No suprathreshold voxels							

^†^ Cluster crosses midline.

#### 3.4.2. Change in functional activity after surgery

During *a priori* ROI analysis, there were clusters of increased BOLD signal after surgery that survived thresholding at *P *< 0.05_FWE_. These were more extensive or bilateral at the lenient thresholds (see Table 5B and Figure 5). During whole brain analysis, there was a small cluster of significantly increased BOLD signal that survived thresholding at *P *< 0.05_FWE_, within the left planum polare.

**Table 5B:** Clusters of increased BOLD signal after surgery within the patient group (visit 1 vs. 2) (significant results shown at each threshold level in order to demonstrate corresponding cluster size).

fMRI results							
Patients: pre- vs. postoperative (visit 1 vs. visit 2)							
***ROI analysis***							
Threshold	ROI	Side	X	Y	Z	*T* Score	*k*
*P *< 0.05_FWE_	ACC	R	4	6	28	4.98	3
	Insula	R	40	22	−6	4.58	1
	OFC	R	22	40	−14	5.05	1
		L	−24	40	−12	5.04	1
*P *< 0.001, ≥10 voxels	ACC	R	4	6	28	4.98	13
	Insula	R	40	22	−6	4.58	12
	OFC	R	22	40	−14	5.05	42
		L	−24	40	−12	5.04	14
*P *< 0.00625, ≥10 voxels	ACC	R	4	6	28	4.98	36
	Insula	R	40	22	−6	4.58	32
	OFC	R	22	40	−14	5.05	57
		L	−24	40	−12	5.04	36
		L	−30	28	−14	4.40	15
		R	42	28	−6	3.82	22
	TP	L	−46	6	−16	3.76	54
***Whole brain analysis***							
Threshold	Region	Side	X	Y	Z	*T* Score	*k*
*P *< 0.05_FWE_	Planum polare	L	−44	−4	−24	6.73	3

#### 3.4.3. Correlation between psychophysical score and BOLD signal

At the exploratory threshold, we demonstrated clusters of significant positive correlation between BOLD signal and composite TDI score as well as individual T and D scores within the right insula (see Table 5C). There were additionally clusters of significant positive correlation between T score and BOLD signal within the right OFC and ACC, though only the former survived thresholding at the cluster criterion. Of note, clusters within the OFC and insula closely neighbour clusters of increased BOLD signal after surgery.

**Table 5C:** Clusters of significant positive correlation between psychophysical test score and BOLD signal.

fMRI results								
Correlation: psychophysical score ∝ BOLD								
***ROI analysis***								
Threshold	Psychophysical score	ROI	Side	X	Y	Z	*T* Score	*k*
*P *< 0.00625, ≥10 voxels	T	OFC	R	22	44	−14	3.48	20
		Insula	R	40	20	6	3.49	129
	D	Insula	R	42	18	−6	2.90	36
	TDI	Insula	R	36	18	4	3.14	89
***Whole brain analysis***								
No suprathreshold voxels								

## 4. Discussion

### 4.1. Neuroanatomical biomarkers of olfactory dysfunction

To our knowledge this is the first prospective study to demonstrate structural and functional plasticity in association with improved olfactory function following fSRP, in patients with non-CRS OD of mixed cause. In each of the *a priori* ROI we demonstrated significant change in GM volume as well as increase in BOLD signal after surgery. Across modalities, results within the ACC and insula were most statistically robust, followed by the OFC and finally the TP. With regards to structural plasticity—we demonstrated reduced GM volume within each of the *a priori* ROIs, as well as a small area of increased GM volume within the OFC. Potentially in line with this, comparison of GM volume in preoperative patients with controls demonstrated areas of reduced GM volume within the OFC, but more widespread areas of increased GM volume within each of the four ROIs. Finally, there was a small cluster of increased BOLD signal after surgery that survived thresholding during whole brain analysis (*P *< 0.05_FWE_) within the left planum polare.

The ACC, insula and OFC are well established nodes within the secondary olfactory network and are frequently activated during functional neuroimaging studies (24) [see (7) for detailed discussion]. Human tractography studies have demonstrated direct connections between the insula and ACC, the insula and OFC, as well as between the ACC and OFC (25, 26). The functional importance of this anatomical connectivity is highlighted by time series in which activations of the ACC, OFC and insula temporally overlap (27, 28). More generally, the anterior insula and dorsal ACC are important nodes within the salience network: a bilateral system that integrates emotional and interoceptive input with external sensory information and which interacts with other neurocognitive networks such as the central executive network and default-mode network (29, 30). It is worth noting that the ventromedial prefrontal cortex (whose boundaries, depending on definition, either overlap or are synonymous with the OFC) is a key node within the default mode network (31, 32). These structural and functional interconnections may underlie the pattern of results we demonstrated in our previous work—in which we demonstrated functionally significant structural plasticity within the ACC, insula and OFC (7)—and which we have replicated in the current study.

We demonstrated less statistically robust results for structural and functional plasticity within the TP. However, we additionally demonstrated increased BOLD signal after surgery within the structurally adjacent left planum polare, which survived thresholding at the whole brain level. Though less well established, the TP is a component of the secondary olfactory network and more generally involved in multimodal sensory integration, particularly in the context of social cognition (33). In humans, anatomical connections between the TP and the insula, OFC and ACC have been demonstrated (25, 34). Part of the superior temporal gyrus (STG), the planum polare neighbours the temporal pole and has known anatomical connections with the insula (25). The STG is also known to have connections with the OFC (26), making these regions highly interconnected. Similar to the TP, the STG is thought to be involved in the hedonic processing of olfactory stimuli (35) and more generally in contextual integration (36). It has also been suggested that the left planum polare and TP may be part of a joint network (which also includes the insula and OFC) that guides behaviour in response to salient olfactory stimuli (37). Our observed increase in BOLD signal after surgery within the left planum polare may therefore be related to the other changes we observed within structures of the salience network. However, this remains speculative at present.

Of interest, we replicated the direction of structural plasticity observed in our earlier study: improved olfactory function appears to be associated with reductions in GM volume. This is in contrast to results demonstrated in patients undergoing olfactory training, where GM volume appears to increase in association with improved olfactory function (38, 39). We previously hypothesised that mechanistic differences may underlie the differences seen: olfactory training may involve a top-down learning process leading to increased GM volume, whilst surgery—through modification of the peripheral olfactory apparatus and thereby increased peripheral input—may involve a bottom-up process leading to reduced GM volume, possibly through improved network efficiency and associated synaptic pruning or other microanatomical changes. In theory, such differences in short-term structural plasticity do not preclude GM atrophy following prolonged reduced afferent input or other central olfactory dysfunction.

This theory for mechanistically divergent improvement in olfactory function is supported by observations that olfactory training improves odour identification more than odour threshold (40), the latter of which is thought to better reflect peripheral olfactory apparatus function (14), as targeted by surgery. Accordingly, it is interesting to note that in our current cohort, clusters of increased BOLD signal after surgery were spatially aligned with clusters of significant positive correlation between BOLD signal and threshold (T) score within the OFC and insula. Taken together, where reduced GM volume and increased BOLD signal are speculated to reflect better network efficiency (caused by increased peripheral sensory input), the anatomical regions involved may implicate changes within networks that modulate attention to olfactory stimuli. In line with this, patients are thought to spend more time attending to odours than healthy controls (41). However, this remains highly speculative and requires investigation with future longitudinal structural, connectivity and task-based functional analyses.

Finally, the differences in directionality in GM volume we demonstrated [when compared both with longitudinal olfactory training work, and cross sectional disease state VBM -see (7)] may also be due to a non-linear time course in structural plasticity or, more simply, due to sampling variation. However, we would suggest, given our replicated demonstration of functionally relevant structural plasticity within the ACC, OFC, insula and TP, that these regions are neuroanatomical correlates of OD, independent of directionality of GM volume change. Furthermore, as we replicated these results in patients with OD of mixed cause undergoing fSRP, the plasticity demonstrated appears to be related to general, rather than disease-specific OD or treatment-specific change in olfaction.

### 4.2. Novel fSRP mechanistic insights

Previous evidence for improved olfaction in functional septorhinoplasty is limited by methodological inconsistencies. A meta-analysis by Pfaff and colleagues demonstrated overall improvement in olfactory function, but studies varied in terms of procedure (functional vs. aesthetic), baseline olfactory function, and outcome measures used (8). Comparison of different outcome measures is particularly problematic: for example, subjective and psychophysical measures are known to correlate poorly, in both patient and healthy participant cohorts (42–44). To our knowledge, this is the first study to demonstrate improved multicomponent psychophysical and patient-reported measures of olfaction, in conjunction with a novel and objective outcome measure—structural and functional plasticity—after fSRP in patients with OD.

Whilst the underlying mechanisms for improvement in olfactory function after fSRP have yet to be fully delineated, our results provide some initial insights. Of interest, we demonstrated significant positive correlation between change in threshold score after surgery and change in bilateral PNIF score. However, we were unable to demonstrate significant positive correlations between change in *AD* or *AS* score and change in psychophysical test scores. It would therefore appear that changes in overall airflow were more physiologically important with respect to olfaction than improved nasal airflow symmetry, following fSRP in our cohort. Previous work using computational fluid dynamics has demonstrated that airflow to the olfactory cleft region is critically affected by anatomical alterations within the olfactory cleft itself, and importantly, the internal nasal valve (“INV”) region (45). As we performed augmentation of the bilateral INV as standard, one may speculate that resultant changes in nasal airflow facilitated odorant access to the olfactory cleft, which was better reflected by changes in bilateral PNIF than measures of airflow symmetry. Increasing odorant access to the OC may improve olfaction in the short-term by increased odorant-olfactory receptor binding and long-term by a putative bottom-up plasticity process induced by improved peripheral input. The latter may be reflected in our neuroimaging findings, where we demonstrated spatial alignment between clusters of increased BOLD signal after surgery and clusters of significant positive correlation between BOLD signal and T score within the OFC and insula.

Whilst our findings require replication in a larger cohort, we would suggest that augmentation of the bilateral INV may be beneficial to olfaction and that future research should aim to investigate this further, in patients both with and without significant septal deviations. Finally, our results may explain the relatively poor evidence for improved olfaction after septoplasty (46), which preferentially corrects symmetry rather than overall nasal airflow.

### 4.3. Study limitations

Three limitations of this work are: (1) small sample size; (2) lack of prospective control arm; (3) mixed aetiology of OD.

Our sample size was determined in relation to our primary neuroimaging aim, with minimum participant number determined from existing literature (47–49), and available pre-pandemic patient population. Statistical power (the probability of rejecting the null hypothesis when it is false) is defined according to: (1) effect size (and its variance); (2) alpha value; (3) sample size. Determination of sample size required to achieve a pre-specified statistical power (e.g., 80%), therefore requires some pre-existing knowledge of effect size. In neuroimaging studies, effect size is the percent signal change between experimental and control conditions. Given the mass univariate approach used in SPM, an effect size can be defined for each individual voxel (of approximately >120,000 voxels for a whole brain analysis), or a mean effect size across a pre-defined cluster of voxels. Furthermore, the variance of the effect size at each voxel/cluster is required—including both intra- and inter-subject variability. With this in mind, most approaches to power calculations in neuroimaging require pilot data (50) or reliance on simulated data (51). Moreover, as we were performing a multimodal neuroimaging study, estimated effect size and its variance at each voxel/cluster of voxels would be required for each modality type (VBM/fMRI/CT), with arbitrary prioritisation of one modality in determining sample size. As this study was itself pilot work, a power calculation was not performed, with minimum participant number taken from the available literature, as is standard practice. However, our results may be used to inform future neuroimaging power analyses, where appropriate. In future work where the primary aim is to investigate the effect of fSRP (i.e., non-neuroimaging primary aim), studies should be accordingly powered using the MCID for the psychophysical tool used (e.g., 5.5 for composite TDI), with appropriate associated control groups.

Whilst our final sample size was comparatively small, we were able to demonstrate significant results using established methods to control for false positives, indicating our respective effect sizes are larger than if the same were demonstrated with a lager sample (52). Whilst a larger sample size may have revealed further significant results and potentially reduced sampling variation, lack thereof does not invalidate the current findings of this pilot study. However, future work should aim to incorporate larger participant numbers.

Lack of prospective control arm is a limitation of the current pilot study. However, our previous work demonstrated these neuroanatomical regions to undergo functionally significant structural plasticity in comparison with a prospective control group (7). We were therefore confident that these regions would not undergo plastic change in the control group. Furthermore, there is precedent for such study design in the neuroimaging literature (48). However, future studies should incorporate a prospective control arm where possible.

Another potential limitation was use of a mixed OD aetiology cohort. As a pilot study with a pragmatic study sample, it was not possible to recruit eligible patients from only one underlying aetiology of OD due to the small available patient population. However, there is extensive precedent for use of mixed aetiology cohorts in the olfactory neuroimaging literature (53). Moreover, as our primary aim was to determine whether functionally significant structural plasticity occurs following treatment of general, rather than aetiology-specific OD, a mixed aetiology study sample was felt to be appropriate. Furthermore, due to the pragmatic study sample, it was not possible to exclude participants based on allergic rhinitis (AR) status. In light of this, participants were carefully screened (clinical history, endoscopy and imaging findings), to exclude CRS, in line with current guidelines (9, 54). To further mitigate the potential effects of AR, and other potentially unknown confounding factors, our control group was taken from a cohort of normosmic patients also awaiting functional septorhinoplasty. Accordingly, there was no significant difference in the proportion of participants with AR in the patient vs. control group (see Table 2). However, larger future studies should aim to exclude patients with AR.

### 4.4. Conclusion

This is the first prospective study to demonstrate structural and functional plasticity in association with improved olfactory function following fSRP, in patients with non-CRS OD of mixed cause. Combined, our work supports the role of the ACC, insula, OFC, and TP as neuroanatomical correlates of OD. In future, the clinical utility of these regions as personalised biomarkers of OD could be explored, as well as the role of fSRP in the treatment of this important sensory impairment.

## Data Availability

The raw data supporting the conclusions of this article will be made available by the authors, without undue reservation.
